# Potentiation of ionising radiation by targeting tumour necrosis factor alpha using a bispecific antibody in human pancreatic cancer

**DOI:** 10.1038/sj.bjc.6601362

**Published:** 2003-11-11

**Authors:** D Azria, C Larbouret, V Garambois, A Kramar, P Martineau, B Robert, N Aillères, M Ychou, J B Dubois, A Pèlegrin

**Affiliations:** 1 Tumour Immunotargeting and Antibody Engineering, INSERM, EMI 0227, 34298 Montpellier, France; 2 Department of Radiation Oncology, Val d'Aurelle Cancer Institute, 34298 Montpellier, France; 3 Biostatistics Unit, Val d'Aurelle Cancer Institute, 34298 Montpellier, France; 4 Center for Pharmacology and Health Biotechnology, CNRS, 34000 Montpellier, France; 5 Department of Radiophysics, Val d'Aurelle Cancer Institute, 34298 Montpellier, France; 6 Department of Medical Oncology, Val d'Aurelle Cancer Institute, 34298 Montpellier, France

**Keywords:** bispecific antibody, tumour necrosis factor alpha, pancreas cancer, radiation enhancement

## Abstract

The aim of this study was to treat carcinoembryonic antigen (CEA)-expressing pancreatic carcinoma cells with tumour necrosis factor alpha (TNF*α*) and simultaneous radiation therapy (RT), using a bispecific antibody (BAb) anti-TNF*α*/anti-CEA. TNF*α* used alone produced a dose-dependent inhibition of the clonogenic capacity of the cultured cells. Flow cytometry analysis of cell cycle progression confirmed the accumulation of cells in G_1_ phase after exposure to TNF*α*. When TNF*α* was added 12 h before RT, the surviving fraction at 2 Gy was 60% lower than that obtained with irradiation alone (0.29 *vs* 0.73, respectively, *P*<0.00001). In combination treatment, cell cycle analysis demonstrated that TNF*α* reduced the number of cells in radiation-induced G_2_ arrest, blocked irreversibly the cells in G_1_ phase, and showed an additive decrease of the number of cells in S phase. In mice, RT as a single agent slowed tumour progression as compared with the control group (*P*<0.00001). BAb+TNF*α*+RT combination enhanced the delay for the tumour to reach 1500 mm^3^ as compared with RT alone or with RT+TNF*α* (*P*=0.0011). Median delays were 90, 93, and 142 days for RT alone, RT+TNF*α*, and RT+BAb+TNF*α* groups, respectively. These results suggest that TNF*α* in combination with BAb and RT may be beneficial for the treatment of pancreatic cancer in locally advanced or adjuvant settings.

Adenocarcinoma of the pancreas remains one of the most difficult malignancies to treat. The incidence has steadily increased over the past four decades (Gudjonsson, 1987), and its prognosis is still dismal, despite tremendous efforts in early diagnosis and therapy. The 5-year survival rate is less than 5% with a complete surgical resection (Gudjonsson, 1987), ranking this cancer fourth among the leading causes of cancer death (Parker *et al*, 1996). Unfortunately, at the time of diagnosis, the majority of patients (80–90%) have locally or metastatic inoperable tumours. Radiation therapy (RT) alone or in combination with chemotherapy showed modest efficacy in local control and palliation (Andre *et al*, 2000; Kornek *et al*, 2000; Azria *et al*, 2002). Despite these intensive efforts to improve the efficacy of conventional therapy, no satisfactory progress in dealing with this cancer has been made. Accordingly, new treatment modalities are required for this tumour.

Current interest has focused on biological response modifiers as antineoplastic agents. Among them, tumour necrosis factor alpha (TNF*α*) was originally identified as a tumoricidal protein effecting haemorrhagic necrosis of transplanted solid tumours in mice (Carswell *et al*, 1975). It is a multipotent cytokine produced mainly by activated macrophages with the ability to mediate cytotoxicity both *in vitro* (Sugarman *et al*, 1985) and *in vivo* (Carswell *et al*, 1975; Helson *et al*, 1979). TNF*α* usually does not kill untransformed cells (Sugarman *et al*, 1985) but shows an antiproliferative effect on certain tumour cells *in vitro* by still undefined mechanisms. Recently, Ruegg *et al* (1998) reported evidence for the involvement of endothelial cell integrin *α*v*β*3 in the disruption of the tumour vasculature induced by the combination of TNF*α* and IFN*γ*.

Several *in vitro* clonogenic assays suggest that an additive or a supra-additive interaction may occur between TNF*α* and ionising radiation (Hallahan *et al*, 1990; Gridley *et al*, 1994a; Azria *et al*, 2003a) as well as an enhancement of the antitumour effect of radiation in some murine and human tumours *in vivo* (Sersa *et al*, 1988; Nishiguchi *et al*, 1990; Gridley *et al*, 1997; Azria *et al*, 2003a). The oxidative damage produced by TNF*α* (Zimmerman *et al*, 1989) may enhance cellular damage produced by ionising radiation. In addition, TNF*α* and radiation can induce apoptosis in target cells (Yamada and Ohyama, 1988; Langley *et al*, 1993) even if cells are normally highly resistant to the induction of radiation-induced apoptosis (Kimura *et al*, 1999).

In different clinical trials using systemic injection of TNF*α*, the results have been disappointing mainly because patients were found to have significantly lower maximum tolerated doses (Abbruzzese *et al*, 1989; Moritz *et al*, 1989) as compared with mice (Asher *et al*, 1987; Havell *et al*, 1988). These limited results were probably due to the short circulatory half-life of TNF*α* and its severe systemic side effects. Studies involving regional (Lienard *et al*, 1992; Mavligit *et al*, 1992; Lejeune *et al*, 1998) or intratumoral (van der Schelling *et al*, 1992) injection of TNF*α* have demonstrated its potential for cancer therapy, but only when a high enough therapeutic concentration of TNF*α* was obtained in the tumour with a nontoxic systemic concentration. To overcome this limitation, we used previously a bispecific antibody (BAb) directed against carcinoembryonic antigen (CEA) and TNF*α* to target this cytokine in human CEA-expressing colorectal carcinoma treated simultaneously with RT (Azria *et al*, 2003a).

In the present study, we report the results of clonogenic tests of pancreatic (BxPC-3) cell survival, which confirm a superiority of the radiation-TNF*α* combination as compared with radiation alone. We show here a nonreversible cell cycle arrest of these cells treated by TNF*α* alone or in combination with ionising radiation. Using nude mice-bearing BxPC-3 xenografts, we showed a significant enhanced tumour growth delay when the BAb+TNF*α*+RT combination was used as compared with RT alone and with RT+TNF*α*.

## MATERIALS AND METHODS

### Cell line and cell culture

The CEA-expressing human pancreatic carcinoma BxPC-3 cell line (Tan *et al*, 1986) was obtained from the American Type Culture Collection (Rockville, MD, USA). The cells were cultured in RPMI-1640 medium (Gibco Laboratories, France) supplemented with 10% heat-inactivated fetal calf serum (Gibco Laboratories, France), 300 *μ*g ml^−1^ glutamine, 0.25 *μ*g ml^−1^ fungizone, 100 *μ*g ml^−1^ streptomycin, and 100 U ml^−1^ penicillin G. These cells were adherent and grew as monolayers at 37°C in a humidified 5% CO_2_ incubator. The BxPC-3 cells were harvested with 0.5 g l^−1^ trypsin (Gibco Laboratories, France) and 0.2 g l^−1^ EDTA (Gibco Laboratories, France) for 3 min. Cultures were checked for the absence of mycoplasma every month.

### TNF*α* and BAb

Recombinant human TNF*α*, kindly provided by Dr GR Adolf (Boehringer Ingelheim, Wien, Austria), was prepared by expression of a synthetic gene in *Escherichia coli*. The specific activity of TNF*α*, determined in the presence of actinomycin D, was 5 × 10^7^ U mg^−1^ protein as determined by cytolysis of murine L929 cells. TNF*α* (at a concentration of 2.5 mg ml^−1^) was stored at –80°C until use.

BAb was constructed as previously described (Robert *et al*, 1996) from the anti-CEA MAb 35A7 (Haskell *et al*, 1983) and the MAb tnf18 kindly provided by Dr M Brockhaus (Hoffmann-La Roche AG, Basel, Switzerland).

### Radiation protocols

Cells were plated in 10 ml RPMI (to ensure homogeneous energy deposition within each dish) using 60-mm Petri dishes and irradiated with a cobalt-60 (^60^Co) source (*γ*-irradiation, ELITE 100, Theratronics) in the Radiation Department. The radiation was delivered as a single dose ranging from 2 to 10 Gy in an 11 cm × 11 cm field size at a dose rate of 0.5 Gy min^−1^. A 3-cm polystyrene block was used under the Petri dishes during each irradiation to allow homogeneous back-scattering *γ*-rays. Source-half depth distance (SHD) was initially calculated to obtain a constant dose rate of 0.5 Gy min^−1^ and monthly adapted from the ^60^Co source radioactivity decrease. Control cells were removed from the incubator and placed for the same period of time under the ^60^Co source but without radiation treatment. In the combined treatment modality studies, TNF*α* was added 12 h prior to RT (see Results, ‘TNF*α* enhances radiosensitivity’).

For *in vivo* tumour treatment, the radiation was delivered to the flank of five mice simultaneously in a 12.5 cm × 12.5 cm field size at 6 Gy fraction^−1^ at a dose rate of 0.5 Gy min^−1^ (SHD of 158 cm), twice a week, for a total dose of 30 Gy. A 6-cm thickness lead block with eight circular apertures, 3 cm in diameter, was used so that only the tumours and the underlying normal tissues were exposed to the radiation. Radiation was measured using dosimetry films (RA711P, Agfa, Belgium).

Immediately prior to irradiation, the mice were anaesthetised by intraperitoneal injection of 233 *μ*g g^−1^ of tribromoethanol dissolved in an ethanol : saline combination (1 : 10, v v^−1^). The anaesthetic was given to all mice, regardless of treatment group, to equalise the effects due to stress.

### Clonogenic assay

The colony-forming assay and growth curve analyses were used to assess the sensitivity of the BxPC-3 cells to TNF*α*. Cultures were trypsinised, washed, and cells were plated in quintuplicate at a density of 100 per 60-mm Petri dishes. TNF*α* was added at concentrations ranging from 0.3 to 5000 U ml^−1^ 12 h after the cells were plated to allow for cell attachment. Cells were incubated at 37°C in a humidified chamber containing 5% CO_2_ for 12 days. The colonies were then fixed with a 1 : 3 (v v^−1^) acetic acid : methanol solution and stained with 10% Giemsa (Sigma Chemical Co., St Louis, MO, USA); colonies of more than 50 cells were scored. Plating efficiency was calculated with and without TNF*α*. The dose–response curves were fitted to a four-parameter logistic model, where the response, *R*, varies with the dose, *D*, according to the equation: *R*=*a*/(1+(*D*/*b*)*^c^*)+*R*, where *a* is the difference between the maximum and minimum response, *b* is the concentration of drug needed to obtain 50% of the maximal effect, *c* is a slope factor, and *R* is the maximal effect. The cytotoxic effect of irradiation on asynchronous, exponentially growing BxC-3 cells was also determined by the colony-forming assay. Before irradiation, cell density was determined using appropriate dilutions (100, 300, 600, and 1600 cells for 0, 2, 4, and 6 Gy, respectively), and five replicates of each dilution were plated in 60-mm Petri dishes. Cells were irradiated as described above, 24 h after plating to allow for cell attachment prior to the administration of radiation. The TNF*α*-containing medium was given at a concentration of 625 U ml^−1^ 12 h before irradiation. A dose of 625 U ml^−1^ of TNF*α* was chosen because colony-forming assays showed that this dose was sufficient to induce only partial (48% survival) cell growth when the cytokine was used alone. Cultures were irradiated when the drug was in the medium and were immediately returned to the incubator after irradiation. Colonies were counted after 14 days. Experimentally derived data points are the mean of three experiments. The multitarget model survival curves were fit to the data using a least-squares regression to the linear-quadratic model, *S*=*S*_0_ exp (−*αD*_1_−*βD*_1_^2^), where *D*_1_ is the radiation dose, *S* the surviving fraction, and *S*_0_ a normalising parameter.

### Flow cytometry

Cells were plated in 60-mm Petri dishes at a density of 5 × 10^6^ cells dish^−1^. Treatment consisted of TNF*α* (625 U ml^−1^) alone at 24 h (H24), RT (4 Gy) at H36, or TNF*α* (625 U ml^−1^ at H24)+RT (4 Gy at H36). Cells were collected at 48 and 96 h after cell culture and processed for cell cycle analysis. Cells were harvested by trypsinisation, washed with PBS, and then 1 × 10^6^ cells dish^−1^ of treatments were fixed in 70% ethanol for 2 min. After removal of ethanol by centrifugation, cells were then stained with a solution containing 40 *μ*g ml^−1^ propidium iodide (Sigma, St Louis, USA) and 0.1 mg ml^−1^ RNase A (Roche, Indianapolis, USA). Stained nuclei were analysed for DNA-PI fluorescence using a Becton Dickinson FACScan flow cytometer. Resulting DNA distributions were analysed by the CellQUEST software (Becton Dickinson, Mountain View, CA, USA) for the proportion of cells in sub-G0, G1, S, and G2–M phases of the cell cycle.

In a second series of experiments, cells were treated with TNF*α* (625 U ml^−1^) alone at H24 and then cultured for 3 days. Medium was then harvested and replaced by RPMI. Cells were stained at different time points up to 21 days and analysed for DNA content on a FACScan as described above.

### *In vivo* model

All the *in vivo* experiments were performed in compliance with the French guidelines for experimental animal studies (Agreement No. A34220) and fulfil the UKCCCR Guidelines for the welfare of animals in experimental neoplasia.

#### Mice

Athymic 7–9-week-old female Swiss nude mice (nu/nu, Iffa Credo, l'Arbresle, France) were housed in self-contained filter-top cages (five mice cage^−1^) in a facility controlled for temperature, humidity, and a 12 : 12 h light : dark cycle under sterile conditions. The animals were given autoclaved food and water *ad libitum*.

#### Experimental protocols

The human pancreatic carcinoma BxPC-3 cells were harvested with 0.25% trypsin solution, washed, and adjusted to 2 × 10^6^ 150 *μ*l^−1^ RPMI-1640 medium without fetal calf serum. Each mouse was injected s.c. in the right flank with 150 *μ*l of the cell suspension. After 35 days, the mice were grouped according to tumour size by measuring tumour diameters with a Vernier caliper to avoid nonhomogeneous groups before beginning treatments. Tumour dimensions were measured twice weekly and volumes (mm^3^) were estimated by the formula *d*_1_ × *d*_2_ × *d*_3_/2, where *d*_1_ is the length, *d*_2_ is the width, and *d*_3_ is the height of the tumour.

On day 35, the mice were assigned to seven different treatment groups (five mice per group) as follows:

*Group* 1: 0.9% NaCl i.v. injection alone (200 *μ*l injection^−1^) for this control group on days 34, 37, 41, 44, and 48.

*Group* 2: TNF*α* at 1 *μ*g i.v.^−1^ injection alone (in 200 *μ*l 0.9% NaCl injection^−1^) on days 34, 37, 41, 44, and 48.

*Group* 3: BAb at 25 *μ*g i.v.^−1^ injection alone (in 200 *μ*l 0.9% NaCl injection^−1^) on days 33, 36, 40, 43, and 47.

*Group* 4: BAb+TNF*α* (ratio 25 *μ*g : 1 *μ*g; molar ratio 12.5 : 1) i.v. injection (in 200 *μ*l 0.9% NaCl injection^−1^) on days 33, 36, 40, 43, and 47. BAb–TNF*α* mixture was prepared 24 h before injection.

*Group* 5: Local radiation as described above delivered on days 34, 37, 41, 44, and 48+0.9% NaCl i.v. injection (200 *μ*l injection^−1^) 3 h before irradiation.

*Group* 6: Local radiation as described above delivered on days 34, 37, 41, 44, and 48+TNF*α* i.v. injection administered using the same time–dose schedules as for group 2 with TNF*α* injections 3 h before irradiation

*Group* 7: Local radiation+BAb+TNF*α* administered using the same time–dose schedules as for group 4 concerning BAb+TNF*α* and group 5 in regard to radiation.

All i.v. injections were performed in the heat dilated tail vein; the day of tumour implantation was day 0. On the basis of the biodistribution studies of TNF*α* and BAb–TNF*α* complexes (Robert *et al*, 1996), we decided to inject TNF*α* 3 h prior to RT (group 6) and BAb–TNF*α* complexes 24 h prior to RT (group 7).

The mice were weighed twice a week and routinely observed for signs of toxicity throughout the study particularly digestive toxicity because of the local flank irradiation.

### Statistical analyses

The nonparametric Wilcoxon's signed-rank test was used to compare the surviving fraction between the two groups (RT alone and RT+TNF*α*). For *in vivo* experiments, the results were expressed in terms of the time taken for the tumour to reach a volume of 1500 mm^3^. The Kaplan–Meier method was used to estimate the median time taken to reach a tumour volume of at least 1500 mm^3^. Differences among treatment groups were tested by the log-rank test. All statistical tests were two-sided with an *α* level of 0.05. Data were analysed with software STATA 7.0 (Stata Corporation, College Station, TX, USA).

## RESULTS

### TNF*α* inhibits BxPC-3 proliferation

The cytotoxic effects of increasing concentrations of TNF*α* (0.3–5000 U ml^−1^) on asynchronous, exponentially growing BxPC-3 cells were determined in colony-forming assays. Cell survival followed a dose–response curve fitted to a four-parameter logistic model as described in Materials and Methods.

**Figure 1:**
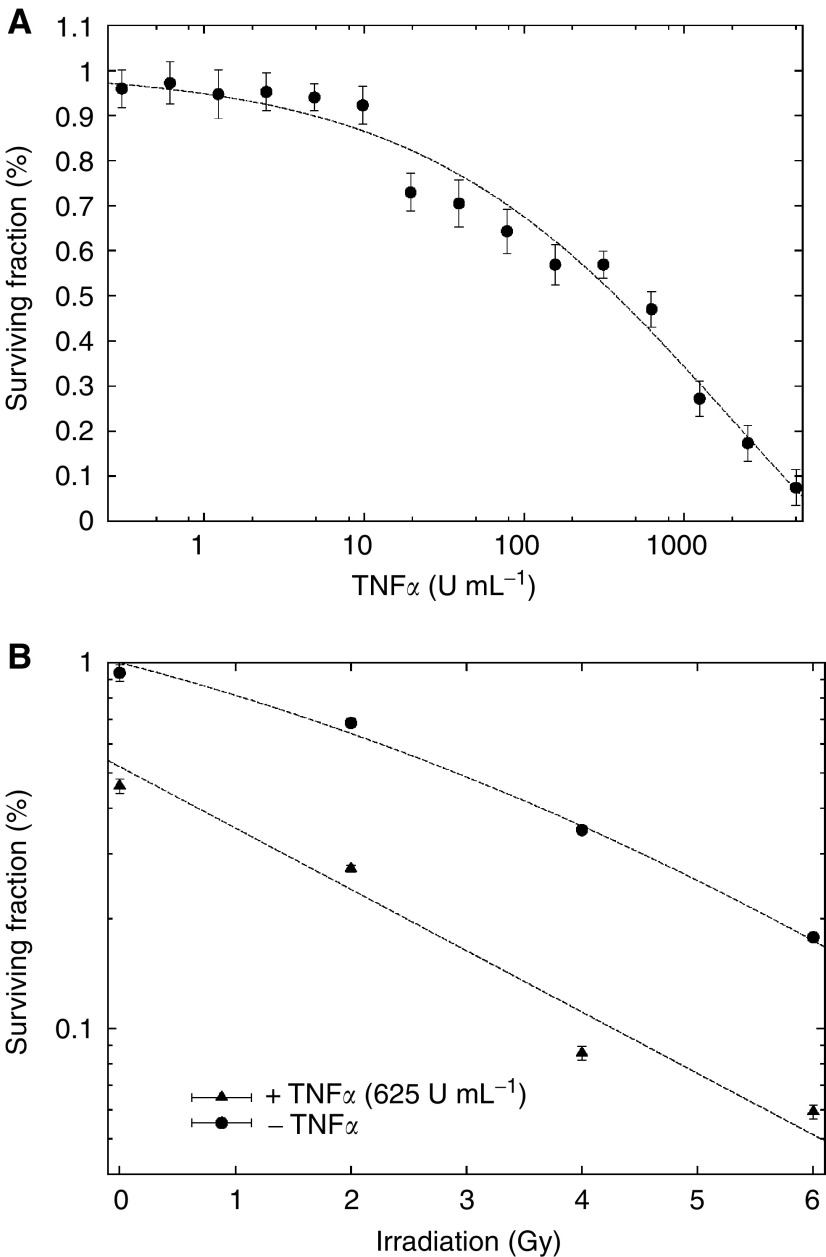
Dose–response curves of the effects of TNF*α* and irradiation treatment of BxPC-3 cells. (**A**) Response of BxPC-3 cells to TNF*α*. Cells were grown in the presence of increasing concentrations of TNF*α* (0.3–5000 U ml^−1^). Plating efficiencies were compared with controls grown without TNF*α* (100% survival). (**B**) Response of BxPC-3 cells, treated or not with TNF*α*, to radiation. BxPC-3 cells treated with TNF*α* (625 U ml^−1^) added 12 h prior to irradiation showed a surviving fraction at 2 Gy, which was statistically lower (*P*<0.00001) than when combination treatment was used. When the data were analysed according to the linear quadratic model, the *α* and *β* components were, respectively, 0.188±0.08 Gy^−1^ and 0.017±0.011 Gy^−2^ without TNF*α* and 0.39±0.06 Gy^−1^ and about 0 Gy^−2^ in combination treatments.

Cells were killed by concentrations of TNF*α* as low as 10 U ml^−1^ (Figure 1A

). The LC_50_, defined as concentration of drug that reduced the cell survival rate to 50% of that of the controls, was 625 U ml^−1^. Next, BxPC-3 cells were treated with TNF*α* (625 U ml^−1^)+BAb (molar ratio of 100 : 1, 1 : 1, or 1 : 100) and plating efficiencies were compared with that obtained with TNF*α* alone. No difference in the surviving fraction was observed when BAb was added to TNF*α* at the same or lower molar ratio. In contrast, when BAb was added in a 100-fold excess, the surviving fraction of cells exposed to 2 Gy was 30% greater than that observed with TNF*α* alone, probably due to competition between the anti-TNF*α* arm of the BAb in solution and the TNF*α* receptor on the cell surface.

### TNF*α* enhances radiosensitivity

**Figure 2:**
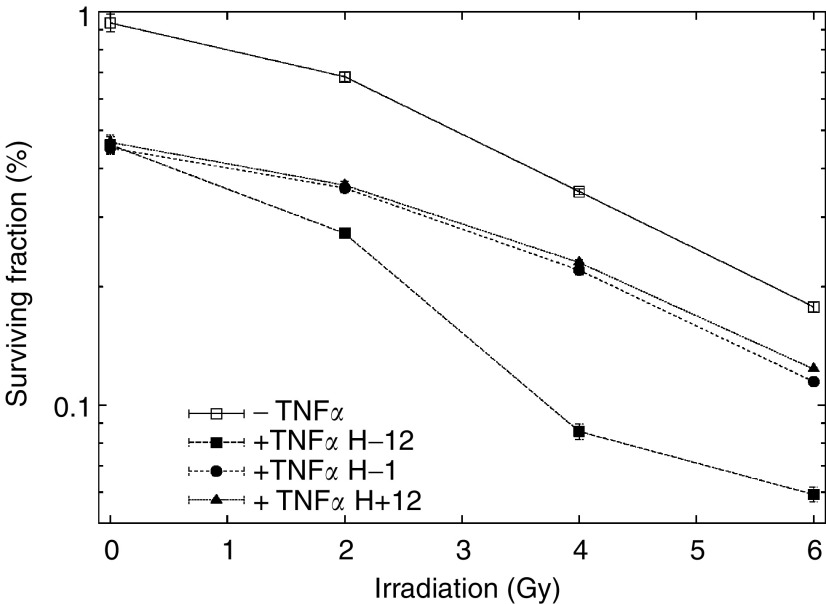
Effect of TNF*α* on the radiosensitivity of BxPC-3 cells. The influence of TNF*α* added 12 h before, 1 h before, or 12 h after RT. Results are expressed in terms of the surviving fraction as described in Materials and Methods. Cells were exposed to TNF*α* (625 U ml^−1^) and irradiated at the different time points. Control cells were exposed to radiation without TNF*α* treatment.

Cell survival following irradiation (Figure 1B) in aerated medium fit a linear quadratic model as described in Materials and Methods. The surviving fraction at 2 Gy (SF2) was 0.73 and a D_0_ (dose of radiation giving 37% survival rate) of 4 Gy when irradiation was used alone. As shown in Figure 2

, TNF*α* added 12 h before RT (H-12) led to a significant decrease of the surviving fractions as compared with those obtained when TNF*α* was added at H-1 or H+12 (*P*=0.02) or when RT was delivered alone. For further experiments using RT with TNF*α*, we used TNF*α* at a concentration of 625 U ml^−1^ added 12 h before RT. In this combination treatment, SF2 and D_0_ were 0.29 and 1.2 Gy, respectively. SF2 was 60% lower in combination treatment with a significant test result (*P*<0.00001). When the data were analysed according to the linear quadratic model, the *α* and *β* components were 0.188±0.08 and 0.017±0.011 Gy^−2^, respectively, without TNF*α* and 0.39±0.06 Gy^−1^ and nearly 0 Gy^−2^ in combination treatments. These data indicate that treatment with TNF*α* results in a steeper decline in cell survival due both to a higher initial slope of the dose–response curve and a major decrease of the quadratic parameter. These results show possible additivity between the two treatments, as confirmed by isobologram analysis (Azria *et al*, 2003b).

### TNF*α* induces G_1_ cell cycle arrest

**Figure 3:**
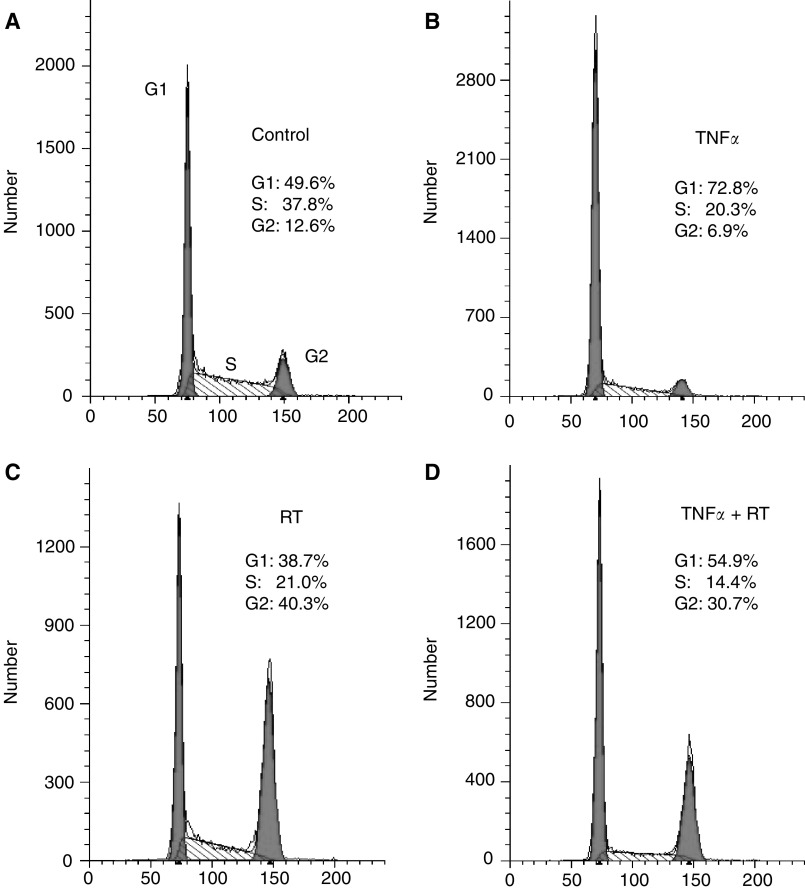
Effect of TNF*α* or/and RT on BxPC-3 cell cycle progression. BxPC-3 were harvested after 36 h exposure to TNF*α* and compared with control cells ((**B**) and (**A**), respectively). In the case of RT treatment, cells were harvested 12 h after RT with or without TNF*α* (added 12 h before RT) (**C** and **D**). Cells were fixed and stained with PI for flow cytometry analysis as described in ‘Materials and Methods.’ Percentages of G_0_/G_1_, S, and G_2_/M were determined by CellQUEST analysis software on the basis of DNA content of the histogram. Data represent mean values of duplicate samples. Similar results were obtained in replicate experiments.

The effect of TNF*α* treatment on cell cycle phase distribution in BxPC-3 cell line was evaluated using flow cytometry (Figure 3

). Treatment with 625 U ml^−1^ TNF*α* for 24 h induced accumulation of cells in G_1_ phase (72.8%) with a significant decrease in the percentage of cells in S phase (20.3%) relative to controls (49.6 and 37.8%, respectively) (Figure 3A and B). No cells with subdiploid DNA content was observed, consistent with other results on human colorectal cell line LS174T (Azria *et al*, 2003b) demonstrating that TNF*α* does not induce apoptosis in these cell lines. After 3 days of treatment, cells were washed and further cultured for 21 days in the absence of the cytokine. We observed a nonreversible G1 cell cycle arrest (nearly 70% at day 21) without any renewal of activity of the S phase as compared to day 3 after TNF*α* treatment (Table 1

**Table 1:** Cell cycle distributions at different times after treatment by TNF*α* in comparison with control (day 0)

**Cycle phases**	**Day 0**	**Day 1**	**Day 3**	**Day 7**	**Day 14**	**Day 21**
G_0_/G_1_	50^b^	73	72	80	78	72
S	38	20	16	12	12	14
G_2_/M	12	7	12	8	10	14

a After 3 days of treatment, cells were washed and further cultured for 21 days in the absence of the cytokine. Cells were fixed and stained with PI for flow cytometry analysis at different time points up to 21 days. DNA histograms were modelled with CellQUEST analysis software and phase percentages for G0/G1, S, and G2/M were determined.

b Results are expressed as mean values (%) of triplicate samples. Similar results were obtained in six independent experiments.

).

At 1 day after RT alone, we observed a cell cycle arrest in the G_2_ phase (40%) with a decrease in the percentage of cells in the G0/G1 and S phases as compared with the control (39 *vs* 50% and 21 *vs* 38%, respectively). When TNF*α* was added 12 h before RT, the radiation-induced G_2_/M arrest decreased as compared with RT alone (31 *vs* 40%, respectively) with a TNF*α*-induced G_0_/G_1_ blockade and a very low S phase (Figure 3C and D).

### BAb+TNF*α* augments *in vivo* tumour response to radiation

BxPC-3 tumours growing s.c. in the right flank of nude mice were used to test the antitumour activity of TNF*α* alone or in combination with RT. TNF*α* was injected i.v. alone or coinjected with the anti-CEA/anti-TNF*α* BAb (BAb–TNF*α* mixture was prepared 24 h before injection at a molar ratio of 12.5 : 1). Median pretreatment tumour volumes (day 35) were 128 (6–135) mm^3^ with no statistical difference between the groups. Tumour growth was then measured regularly until tumours were larger than 1500 mm^3^. Radiation alone (group 5), but not TNF*α* alone (group 2), significantly inhibited tumour progression as compared with the control group (*P*<0.00001). No difference in growth delay was observed between the control group and groups without RT (groups 2–4). During the same period of observation, treatment with TNF*α* slowed tumour growth in irradiated groups, particularly when TNF*α* was coinjected with BAb. At day 93, when mice in all other groups were killed (tumour >1500 mm^3^), the median value of the tumour volume was 260 mm^3^ for the RT+BAb+TNF*α* group.

**Figure 4:**
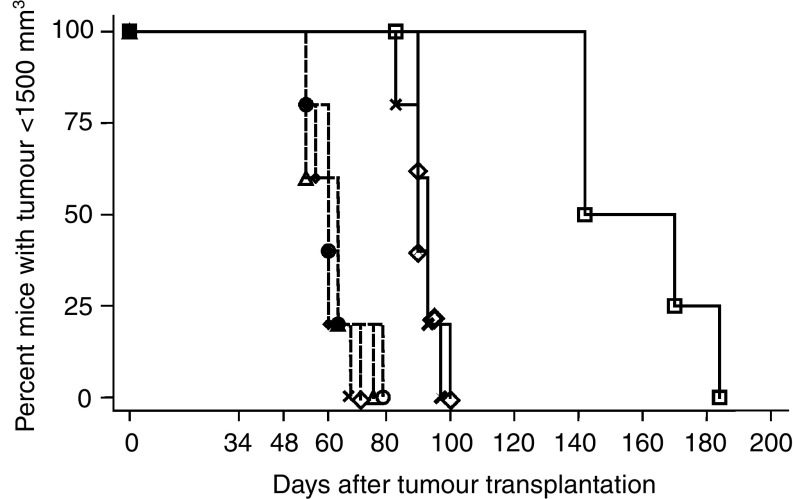
Kaplan–Meier survival curves obtained as a function of time for all groups: group 1: dotted line (○) no treatment (62 days); group 2: dotted line (⋄) TNF*α* (62 days); group 3: dotted line (X) BAb (65 days); group 4: dotted line (△) BAb+TNF*α* (62 days); group 5: solid line (X) RT (90 days); group 6: solid line (⋄) RT+TNF*α* (93 days); group 7: solid line (□) RT+TNF*α*+BAb (142 days). The number in parentheses corresponds to the median delay (time taken for the tumour to reach a volume of 1500 mm^3^ in 50% of the mice).

The results expressed in terms of the time to reach 1500 mm^3^ are shown in Figure 4

. In the control group and the groups treated with TNF*α*, BAb, or BAb+TNF*α*, the median delay for the mice to reach a tumour volume greater than 1500 mm^3^ was 62, 62, 65, and 62 days, respectively, with no statistical difference between the groups. In the RT-treated groups, the median delays were 90, 93, and 142 days for the RT alone, the RT+TNF*α*, and the RT+BAb+TNF*α* groups, respectively. No statistical difference was observed between the RT and RT+TNF*α* groups. However, in the presence of the BAb, the curve for group 7 was shown to be statistically different from the growth curves for tumours treated with RT alone or RT+TNF*α* (*P*=0.0011).

At the end of all treatments, no significant differences were found in mouse body weight between the seven groups. The mean±s.e.m. were 23.1±0.47, 22.4±0.87, 23.6±0.61, 24±0.65, 24±0.37, 24.4±0.41, 23.7±0.54 for groups 1, 2, 3, 4, 5, 6, 7, respectively. No diarrhoea was observed in any group, suggesting the absence of digestive toxicity. No significant fluid retention, respiratory distress, or other signs of toxicity were observed in any of the animals during the course of the study.

## DISCUSSION

Pancreatic carcinoma is the fourth leading cause of cancer deaths. Patient survival of this devastating disease is bleak with less than 5% of patients surviving 5 years after the time of diagnosis (Greenlee *et al*, 2000). The current treatment includes a combination of surgery, chemotherapy, and radiation without any major improvement in survival (Azria *et al*, 2002). Over 10 years ago, it was hypothesised that TNF*α* could increase tumour response to radiation through stimulation of the host antitumour immune responses, direct tumour-cell kill, or through the increase in tumour-cell sensitivity to radiation (Sersa *et al*, 1988; Hallahan *et al*, 1990; Gridley *et al*, 1994a,1994b; Kimura *et al*, 1999; Azria *et al*, 2003a). However, early clinical trials were generally disappointing, with hypotension and vascular leakage frequently being the dose-limiting side effects (Chapman *et al*, 1987; Sherman *et al*, 1988). To overcome these limitations, we used a BAb directed against CEA and human TNF*α* to target this cytokine to the human pancreatic carcinoma cells BxPC-3 treated simultaneously with RT.

In the first part of our study, we demonstrated direct cytotoxicity of TNF*α* on BxPC-3 cells in culture using a clonogenic assay: TNF*α*-treated BxPC-3 cells showed reduced plating efficiency (Figure 1), confirming that TNF*α* can be tumoristatic or tumoricidal as described for a variety of neoplastic cell types (Hallahan *et al*, 1990; Manetta *et al*, 1990; Gridley *et al*, 1994a; Kimura *et al*, 1999; Azria *et al*, 2003a). In a time-course experiment (Figure 2), we demonstrated that maximal cell killing increase was obtained when TNF*α* was added to the cells 12 h before RT as compared with 1 h before and 12 h after RT. These data confirmed those published by Hallahan *et al* (1990), who demonstrated that addition of TNF*α* 4 to 12 h prior to irradiation maximally increases cell killing.

We also observed that TNF*α* induced a G_1_ cell cycle arrest and that cell exposure for 24 h to TNF*α* was sufficient to obtain this effect, which could be considered as irreversible since the G_1_ arrest was maintained up to 21 days after elimination of TNF*α* from the culture medium (Table 1). This effect can probably be explained by modifications of the expression of cell-cycle-related proteins (ongoing research), as described for other cytokine such as interferon *γ* (Matsuoka *et al*, 1999; Gooch *et al*, 2000), and by the fact that TNF*α* induces BxPC-3 cycle distribution modification which may render the cells more radiosensitive. In the RT–TNF*α* combination treatment, we observed a 25% decrease of BxPC-3 cells arrested in the G_2_ phase as compared with RT alone, a proportional redistribution in the G_1_ phase, and an interrupted synthesis phase. We did not observe any induction of apoptosis in BxPC-3 cells, as previously suggested in another model (Gridley *et al*, 1994a) and recently described in a human prostate carcinoma cell line (Kimura *et al*, 1999). This cell cycle redistribution phenomenon may also explain the decrease in the surviving fraction in the combination treatment presented in the present study (Figure 1B). To our knowledge, these results are the first to confirm that TNF*α* is a biological cell cycle modifier, which is responsible for a cell cycle redistribution in the more radiosensitive (G_1_) phase rather than in the S phase. Recently, Dormond *et al* (2002) described that TNF*α* alone or in combination with IFN*γ* induced a G_1_ arrest in endothelial cells (HUVEC), which was associated with reduced levels of cyclin D1 and cdk2, and with increased expression of the cdk inhibitors p16^INK4a^, p21^WAF^, and p27^Kip1^.

In the present study, the *in vitro* growth-inhibitory effect of TNF*α* was accompanied by a marked enhancement of the radioresponse of the tumour *in vivo*, particularly, when TNF*α* was concentrated in the tumour xenografts thanks to our BAb. In addition to the *in vitro* cytotoxic effect of TNF*α*, indirect *in vivo* mechanisms could be responsible for this synergistic rather than additive effect of the combination (Ruegg *et al*, 1998). Several studies have demonstrated the antitumour activity of RT+TNF*α*, but this treatment was given before the tumours reached a palpable volume, making a comparison with our results difficult (Gridley *et al*, 1994b,^1997^). In mammary carcinoma and sarcoma models, TNF*α* was shown to significantly increase tumour radiocurability even when TNF*α* was injected 3 h after RT (Sersa *et al*, 1988; Nishiguchi *et al*, 1990). Our data demonstrate the interest of targeting TNF*α* to tumours to improve RT and finally to keep a large differential effect between tumour and normal tissues. Various methods have recently tried to concentrate TNF*α* into tumour such as Cu2+-dextran (Tabata *et al*, 1999), TNF*α*-biotin conjugates (Moro *et al*, 1997; Gasparri *et al*, 1999), or liposomal encapsulated-TNF*α* (Kim *et al*, 2001) which are less specific targeting than our BAb and were not tested with concomitant radiotherapy.

Another approach currently in clinical evaluation uses an adenoviral vector that contains radio-inducible DNA sequences from the early growth response gene (EGR1) promoter and cDNA for the gene encoding human TNF*α*. While avoiding the systemic side effects of TNF*α*, this method involves injections in or near the tumour, which might be difficult to perform in the case of pelvic or retroperitoneal tumours (Weichselbaum *et al*, 2002).

Concerning the immunotargeting strategy, two attractive methods have been recently described. Cooke *et al* (2002) tested a genetic fusion of human recombinant TNF*α* with MFE-23, a single-chain Fv antibody fragment directed against CEA. Radiolabelled fusion protein binds both human and mouse TNF receptor 1 *in vitro* and *in vivo* and is able to localise effectively in nude mice-bearing human LS174T xenografts with a tumour/tissue ratios of 21 : 1 and 60 : 1 achieved 24 and 48 h after i.v. injection, respectively. The maximum % injected dose (ID) g^−1^ LS174T tumour (4.33) was obtained 6 h postinjection. At that time, in T380 human colon carcinoma nude mice, our BAb was able to concentrate up to 7.15% ID g^−1^ of tumour as compared to 2.2% when BAb was injected alone (Robert *et al*, 1996). Wüest *et al* (2002) described a TNF*α* fusion protein designated TNF-Selectokine, which is a homotrimeric molecule comprised of a single-chain antibody (scFv) targeting molecule, a trimerisation domain and TNF*α*. Membrane targeting dependent immobilisation of this TNF-Selectokine induced cell death in TNFR1 and TNFR2 dependent manner. The authors constructed, also, a TNF-Selectokine prodrug by insertion of a TNFR1 fragment separated from TNF by a protease-sensitive linker in order to restrict TNF activity to the tumour. Both studies suggest interests but are in the early phase of development without any indications of their capacity of radiation enhancement.

The results of our study should be of potential clinical interest. They provide a rational for the combination of TNF*α*, BAb, and RT in the treatment of adenocarcinoma of the pancreas. One of the advantages of our BAb strategy, namely, the potential decrease of TNF*α* systemic toxicity, cannot be addressed in our nude mice model, which lacks T cells. The difference between the TNF*α* +RT and the BAb+TNF*α*+RT combination treatments will probably be even more evident in an immunocompetent model or in a clinical setting. Such an immunocompetent situation is also needed for the entire expression of TNF*α* antitumour action, including immunological (production of IL-1 and IFN*γ*, activation of macrophages, and NK cells; Dinarello *et al*, 1986; Ostensen *et al*, 1987; Talmadge *et al*, 1987) and nonimmunological mechanisms such as damage to the tumour vasculature (Gamble *et al*, 1985; Sato *et al*, 1986; Cavender *et al*, 1987; Ruegg *et al*, 1998).

In conclusion, we demonstrated that an anti-CEA/anti-TNF*α* BAb can markedly enhance the radioresponse of pancreatic tumour xenografts in nude mice. Presently, we are testing the antitumour effect of BAb, TNF*α*, and RT combination in an immunocompetent CEA-transgenic mice transplanted with a syngenic CEA-expressing tumour in which all the effects of the targeted cytokine can be analysed. The next step will be the opening of a phase I clinical study in locally advanced pancreatic cancer.
